# Supplementary material for: The quantum Zeno and anti-Zeno effects with driving fields in the weak and strong coupling regimes
Source: Sci Rep. 2021 Jan 19;11:1836. doi: 10.1038/s41598-021-81424-z (PMC7815882; doi:10.1038/s41598-021-81424-z)
Supplement: Supplementary file 1 — Supplementary Information 1 [file 41598_2021_81424_MOESM1_ESM.pdf]

Supplemental Material for 'The quantum Zeno and anti-Zeno effects with driving fields in the weak and strong coupling regimes'

Mehwish Majeed[1] and Adam Zaman Chaudhry[1,*]

[1]*School of Science & Engineering, Lahore University of Management Sciences (LUMS), Opposite Sector U, D.H.A, Lahore 54792, Pakistan*

In this supplemental Material, we use same symbols as introduced in our main text. For completeness, we present the analytical expressions for the Hamiltonian in the polaron frame. We also outline the calculation of the decay rate $\Gamma(\tau)$ for a single two-level system interacting strongly with an environment of harmonic oscillators.

A. Spin-boson Hamiltonian in polaron frame

To transform spin-boson Hamiltonian to polaron frame, we need to find

$$H^{(P)}(t) = e^{\chi\sigma_z/2} H(t) e^{-\chi\sigma_z/2}.$$

We use the Hadamard lemma

$$e^{A} O e^{-A} = O + [A, O] + \frac{1}{2!}[A, [A, O]] + ... \quad (1)$$

where $A = \chi\sigma_z/2$, with $\chi = \sum_k [\frac{2g_k}{\omega_k} b_k^\dagger - \frac{2g_k^*}{\omega_k} b_k]$ and $O = H(t) = \frac{\varepsilon(t)}{2}\sigma_z + \frac{\Delta}{2}\sigma_x + \sum_k \omega_k b_k^\dagger b_k + \sigma_z \sum_k (g_k^* b_k + g_k b^\dagger)$.

We find that

$$e^{\chi\sigma_z/2}\sigma_z e^{-\chi\sigma_z/2} = \sigma_z,$$

and

$$e^{\chi\sigma_z/2}\sigma_x e^{-\chi\sigma_z/2} = \sigma_+ e^{\chi} + \sigma_- e^{-\chi}.$$

σ_- and σ_+ are the standard spin-1/2 lowering and raising operators. Carrying on further, we find

$$e^{\chi\sigma_z/2}(\sum_k \omega_k b_k^\dagger b_k) e^{-\chi\sigma_z/2} = \sum_k \omega_k b_k^\dagger b_k - \sigma_z \sum_k (g_k^* b_k + g_k b^\dagger) + \sum_k \frac{|g_k|^2}{\omega_k}.$$

* adam.zaman@lums.edu.pk

Similarly

$$e^{\chi\sigma_z/2}\bigg(\sigma_z\sum_k(g_k^* b_k + g_k b^\dagger)\bigg)e^{-\chi\sigma_z/2} = \sigma_z\sum_k(g_k^* b_k + g_k b^\dagger) - 2\sum_k\frac{|g_k|^2}{\omega_k},$$

The third term of Eq. (1) in the above expression is a constant number, so higher order commutators are zero. Now putting all these terms back together, the required Hamiltonian in polaron frame takes the following form

$$H^{(P)}(t) = \frac{\varepsilon(t)}{2}\sigma_z + \sum_k \omega_k b_k^\dagger b_k + \frac{\Delta}{2}(\sigma_+ Y + \sigma_- Y^\dagger) - \sum_k\frac{|g_k|^2}{\omega_k},$$

with $Y = e^\chi$, and $\sum_k \dfrac{|g_k|^2}{\omega_k}$ is a constant number term that gives a constant shift in transformed Hamiltonian, and can thus be dropped.

B. Effective decay rate of spin-boson model in polaron frame

Since system-environment coupling is weak in the polaron frame, we can use expression [see the main text]

$$\Gamma(\tau) = \frac{2}{\tau}\mathrm{Re}\bigg(\sum_{\mu\nu}\int_0^\tau dt\int_0^t dt' C_{\mu\nu}(t')\mathrm{Tr}_S\big[P_\perp \widetilde{F}_\nu(t-t')\rho_S(0)\widetilde{F}_\mu(t)\big]\bigg),$$

to calculate effective decay rate . Here $\rho_S(0) = |e\rangle\langle e|$ and $P_\perp = |g\rangle\langle g|$. We identity $F_1 = \dfrac{\Delta}{2}\sigma_+$, $F_2 = \dfrac{\Delta}{2}\sigma_-$, $B_1 = Y$, $B_2 = Y^\dagger$, $\widetilde{F}_1(t) = \dfrac{\Delta}{2}\sigma_+ e^{i\zeta(t)}$ and $\widetilde{F}_2(t) = \dfrac{\Delta}{2}\sigma_- e^{-i\zeta(t)}$ with $\zeta(t) = \int_0^t dt'\varepsilon(t')$ leading us to

$$\Gamma(\tau) = \frac{2}{\tau}\mathrm{Re}\bigg(\int_0^\tau dt\int_0^t dt' C_{12}(t')e^{i(\zeta(t)-\zeta(t-t'))}\bigg). \tag{2}$$

To get expression of effective decay rate, the environment correlation function $C_{12}(t)$ needs to be worked out. We now show the details how to find C_{12}. As we know $C_{12}(t) = \mathrm{Tr}_B[\rho_B \widetilde{B}_1(t)B_2]$, with $B1 = Y$, $B_2 = Y^\dagger$, $\widetilde{B}_1(t) = e^{iH_B^{(P)}t}Ye^{-iH_B^{(P)}t}$, $H_B^{(P)} = \sum_k \omega_k b_k^\dagger b_k$, $Y = e^\chi$ and $\chi = \sum_k[\dfrac{2g_k}{\omega_k}b_k^\dagger -$

$\dfrac{2g_k^*}{\omega_k}b_k]$. Next, we calculate

$$\widetilde{B}_1(t) = e^{\sum_k \left(\frac{g_k}{\omega_k} b_k^\dagger e^{i\omega_k t} - \frac{g_k^*}{\omega_k} b_k e^{-i\omega_k t}\right)},$$

using the fact $U^\dagger(t) e^A U(t) = e^{U^\dagger(t) A U(t)}$, and then find

$$\widetilde{B}_1(t) B_2 = e^{-i\sum_k \frac{|g_k|^2}{\omega_k^2}\sin(\omega_k t)} e^{\sum_k \left(\frac{g_k}{\omega_k} b_k^\dagger (e^{i\omega_k t}-1) + \frac{g_k^*}{\omega_k} b_k (e^{-i\omega_k t}-1)\right)}.$$

In order to convert double exponential in a single exponential to use useful fact $\mathrm{Tr}_B[\rho_B e^C] = e^{\langle C^2\rangle/2}$, where operator C is a linear combination of annihilation and creation operators; we use the identity $e^X e^Y = e^{X+Y+\frac{1}{2}[X,Y]}$. Fortunately in this case, first commutator is a constant number, so higher order commutators are zero. Finally we have

$$C_{12}(t) = e^{-i\Phi_I(t)} e^{-\Phi_R(t)}.$$

where $\Phi_I(t)$ and $\Phi_R(t)$ have been defined in the main text. Carrying on further,we have

$$\Gamma(\tau) = \frac{\Delta^2}{2\tau}\int_0^\tau dt \int_0^t dt' e^{-\Phi_R(t')} \cos[\zeta(t) - \zeta(t-t') - \Phi_I(t')]. \quad (3)$$
